# Status quo and future directions of digitalization in gynecology and obstetrics in Germany: a survey of the commission Digital Medicine of the German Society for Gynecology and Obstetrics

**DOI:** 10.1007/s00404-023-07222-2

**Published:** 2023-09-27

**Authors:** André Pfob, Christoph Hillen, Katharina Seitz, Sebastian Griewing, Sven Becker, Christian Bayer, Uwe Wagner, Peter Fasching, Markus Wallwiener, For the Kommission Digitale Medizin, Deutsche Gesellschaft für Gynäkologie und Gebursthilfe (DGGG)

**Affiliations:** 1 Department of Obstetrics & Gynecology, Heidelberg University Hospital, Heidelberg, Germany; 2 National Center for Tumor Diseases (NCT), German Cancer Research Center (DKFZ), Heidelberg, Germany; 3 Department of Gynecology and Gynecologic Oncology, University Medical Center Hamburg-Eppendorf, Hamburg, Germany; 4 University Hospital Erlangen, Comprehensive Cancer Center Erlangen-EMN, Department of Gynecology and Obstetrics, Friedrich-Alexander University Erlangen-Nuremberg, Erlangen, Germany; 5 Department of Gynecology and Obstetrics, University Hospital Marburg, Philipps-University Marburg, Baldingerstraße, 35043 Marburg, Germany; 6 Department of Gynecology, University Hospital, Frankfurt am Main, Germany; 7 WMC HEALTHCARE GmbH, München, Germany

**Keywords:** eHealth, Digital health, Artificial intelligence, Implementation, Gynecology and obstetrics

## Abstract

**Purpose:**

Digitalization plays a critical role and is beginning to impact every part of the patient journey, from drug discovery and data collection to treatment and patient-reported outcomes. We aimed to evaluate the status quo and future directions of digital medicine in the specialty of gynecology and obstetrics in Germany.

**Methods:**

An anonymous questionnaire was distributed via the German Society of Gynecology and Obstetrics newsletter in December 2022. The questionnaire covered the domains baseline demographic information, telemedicine, digital health applications (DIGAs), and future expectations.

**Results:**

In all, 91 participants completed the survey. Median age was 34 years; 67.4% (60 of 89) were female and 32.6% (29 of 89) were male. About 10% (9 of 88) have prescribed DIGAs to date and 14% (12 of 86) offer telemedical appointments. Among those who do not use digital medicine, very few plan to do so in the near future. Reasons include missing software interfaces, lack of time to try out new things, lack of knowledge, lack of monetary compensation (66.3%), and employee concerns. A majority agreed that digitalization will help to save time and improve patient care and that intelligent algorithms will aid clinicians in providing patient care to women.

**Conclusions:**

The status quo and future directions of digital medicine in gynecology and obstetrics in Germany are characterized by contradicting expectations regarding the benefits of digital medicine and its actual implementation in clinical routine. This represents an important call to action to meet the requirements of modern patient care.

## What does this study add to the clinical work?

**Table:** 

The status quo and future directions of digital medicine in gynecology and obstetrics in Germany are characterized by contradicting expectations regarding the benefits of digital medicine and its actual implementation in clinical routine.	

## Background

In many areas of medicine, digitalization plays a critical role and is beginning to impact nearly every part of the patient journey, from drug discovery and data collection to treatment and patient-reported outcomes [1–6]. Digital medicine includes a variety of fields, including digital health and care applications (DiGAs), telemedicine, mobile(m)Health solutions/wearable devices, artificial intelligence (AI)/data science, and structured data capture and management systems. Digital medicine could aid in developing innovative treatment concepts by providing individualized therapy and prevention recommendations on the basis of structured, multi-modal data analyses. Digital medicine also holds tremendous potential with respect to reducing the workload of the healthcare workforce by shortening standardized processes [7–9].

Implementation of digital medicine varies across countries and specialties. For example, in fields such as pathology or radiology, AI-assisted algorithms are already being used to improve diagnosis from histopathological and radiological findings [7, 10–12]. Furthermore, telemedicine has already become an important aspect of medical care on an international level. The global telemedicine market reached 56.2 billion USD in 2020 and is expected to reach 175.5 billion by 2026; the leading countries here are China and the United States [13]. Dermatology is one of the leading specialities in the use of telemedicine. In this speciality, video consultations work well and are financially rewarding for practitioners. Thus, when speaking of digital medicine in general, we neglect the specific needs and requirements of each specialty at a given location.

Little is known about the current status and future trends of digital medicine in the medical field of gynecology and obstetrics in Germany. In light of these developments, to complement the importance of digitalization and to build up expertise, the German Association of Gynecology and Obstetrics (DGGG) has launched the “Commission for Digital Medicine”, which is devoted to accelerating the digitalization of gynecological and obstetric care in a collaborative working group; this group aims to promote interuniversity collaboration to tackle present and future challenges. A departure point was set by conducting a qualitative survey to assess the status quo of digitalization within the gynecology and obstetrics community in Germany.

## Materials and methods

### Survey participant recruitment and selection

A link to an anonymous questionnaire was distributed via the society newsletter to members of the German Society of Gynecology and Obstetrics (DGGG, Deutsche Gesellschaft für Gynäkologie und Geburtshilfe) in December 2022. Survey responses were collected and saved anonymously using Google forms. The research was conducted in accordance with the precepts established by the Helsinki Declaration.

### Survey questionnaire

The survey questionnaire consisted of questions determined by an expert panel within the commission with respect to the current status and future trends of obstetrics and gynecology in Germany. The survey covered the domains baseline demographic information, telemedicine, digital health applications (DIGAs), robotic surgery, and future expectations. The final questionnaire was confirmed during an in-person meeting with the whole commission Digital Medicine of the German Society for Gynecology and Obstetrics on July 1, 2022.

### Statistical analysis

Survey responses were analyzed descriptively using absolute values and relative frequencies. Statistical analyses were performed using R software (R Foundation for Statistical Computing).

### Role of the funding source

There was no funding source for this analysis and manuscript.

## Results

### Demographics of survey participants

In all, 91 participants completed the survey. Of those 91 individuals, median age was 34 years and 67.4% (60 of 89) were female, 32.6% (29 of 89) male. With respect to their current working positions, 47.7% (42 of 88) were residents, 26.1% (23 of 91) senior physicians, and 9.1% (8 of 88) medical directors. Further details are shown in Table 1.

**Table 1:** Baseline characteristics of survey participants

Characteristic	Value
Age—yr	
Median (25th, 75th percentile)	34.0 (30.0, 46.25)
Gender—no. (%)	
Female	60 (67.4)
Male	29 (32.6)
No answer	2
Working position—no. (%)	
Resident (Assistenz*ärztin)	42 (47.7)
Attending (Fach*ärztin)	10 (11.4)
Senior physician (Ober*ärztin)	23 (26.1)
Medical director (Chef*ärztin)	8 (9.1)
Physician in private practice (Niedergelassener Fach*ärztin)	3 (3.3)
Honorarium physician (Honorar*ärztin)	2 (2.2)
No answer	3
Worksite—no. (%)	
University hospital	38 (43.2)
Maximum care hospital	8 (9.1)
Medium care hospital	14 (15.9)
Basic care hospital	16 (18.2)
Specialized hospital	4 (4.5)
Private practice	8 (9.1)
Missing	3

### Digital self-assessment

**Fig. 1:**
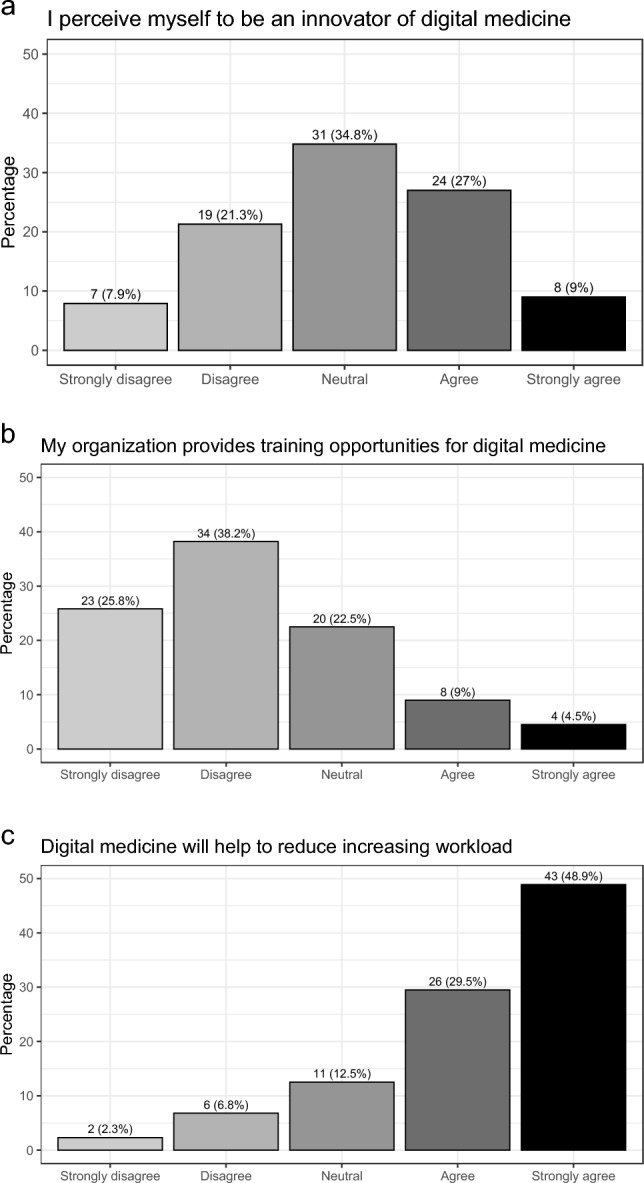
Digital self-assessment of survey participants

As part of a digital self-assessment (Fig. 1), 36% (32 of 89) agreed or strongly agreed that they perceive themselves as innovators for digital medicine, 78.4% (69 of 88) agreed or strongly agreed that digital medicine can help reduce the increasing workload, and only 13.5% (12 of 89) agreed or strongly agreed that their organization provides training for digital medicine.

### Status quo

Participants’ responses regarding the current status quo of digitalization in obstetrics and gynecology in Germany are summarized in Table 2. With respect to DIGAs, about 10% (9 of 88) have prescribed DIGAs so far. Among those who currently do not prescribe DIGAs, only 14.3% are planning to prescribe DIGAs in the future. Reasons for not prescribing DIGAs include lack of knowledge about DIGAs (42.5%), complicated prescription process (23.3%), lack of monetary compensation (17.8%), and lack of evidence (11.0%). Among those who currently prescribe DIGAS, the median number of prescribed DIGAs was 1 (25th percentile 1; 75th percentile 2). Gynecologic oncology (including senology) was the specialty deemed to have the highest potential for DIGAs by those who have not prescribed DIGAs yet (65.7%) as well as by those who have already prescribed DIGAs (77.8%).

**Table 2:** Current status of digitalization in obstetrics and gynecology in Germany

Characteristic	Value
Possibility of performing robotic surgery—no. (%)	
Yes	29 (32.6)
No	60 (67.4)
No answer	2
Number of days per week with possibility to perform robotic surgery—median (25th, 75th percentile)	1 (1, 2.5)
Willingness to participate in app-based trials for rare disease (e.g., granulosa cell tumors)—no. (%)	
Yes	71 (80.7)
No	17 (19.3)
No answer	3
Performing studies in the area digital medicine in obstetrics and gynecology—no. (%)	
Yes	16 (18.0)
No	73 (82.0)
No answer	2)
Number of known medical apps—median (25th, 75th percentile)	
Digital companion apps	2 (0, 3)
Decision support apps	2 (0, 3)
Number of medical apps used personally—median (25, 75 percentile)	2 (0, 3)
Personal use of medical wearables—no. (%)	
Yes	26 (29.5)
No	62 (70.5)
No answer	3
Prescription of DIGAs (Digital Health Applications)—no. (%)	
Yes	9 (10.2)
No	79 (89.8)
No answer	3
Among those who currently do not prescribe DIGAs	
Planning to prescribe DIGAs (Digital Health Applications) in the future among those who currently do not prescribe them—no. (%)	
Yes	11 (14.3)
No	66 (85.7)
No answer	2
Reasons for not prescribing DIGAs—no. (%.)	
Did not know about it	31 (42.5)
Complicated prescription process	17 (23.3)
Lack of monetary compensation	13 (17.8)
Lack of evidence	8 (11.0)
Specialty with highest potential for DIGAs—no. (%.)	
Gynecologic oncology (including senology)	46 (65.7)
Fertility and endocrinology	26 (37.1)
Obstetrics	26 (37.1)
Others	2 (2.8)
No answer	9
Among those who currently prescribe DIGAs	
Number of prescribed DIGAs—median (25th, 75th percentile)	1 (1, 2)
Specialty with highest potential for DIGAs—no. (%.)	
Gynecologic oncology (including senology)	7 (77.8)
Fertility and endocrinology	1 (11.1)
Obstetrics	1 (11.1)
Obesity	2 (22.2)
Vaginismus	1 (11.1)
Kind of prescribed DIGA—no. (%.)	
Digital companion	5 (62.5)
Digital monitoring	5 (62.5)
Wearables	1 (12.5)
Offers telemedical appointments—no. (%)	
Yes	12 (14.0)
No	74 (86.0)
No answer	5
Among those who currently do not offer telemedical appointments	
Planning to offer telemedical appointments in the future among those who currently do not prescribe them—no. (%)	
Yes	9 (12.7)
No	62 (87.3)
No answer	3
Reasons for not offering telemedical appointments—no. (%.)	
Did not know about it	11 (16.7)
Complicated prescription process	16 (24.2)
Lack of monetary compensation	19 (28.8)
Lack of evidence	7 (10.6)
Specialty with highest potential for telemedical appointments—no. (%.)	
Gynecologic oncology (including senology)	43 (62.3)
Fertility and endocrinology	33 (47.8)
Obstetrics	11 (15.9)
Others	3 (4.2)
No answer	5
Among those who currently offer telemedical appointments	
Frequency of telemedical appointments—no. (%.)	
Very often	1 (8.3)
Often	4 (33.3)
Sometimes	6 (50)
Rarely	1 (8.3)
In which specialty do you offer telemedical appointments—no. (%.)	
Gynecologic oncology (including senology)	8 (66.7)
Fertility and endocrinology	5 (11.7)
Obstetrics	3 (25)
Psychotherapy	1 (8.3)

With respect to telemedical appointments, about 14% (12 of 86) offer telemedical appointments. Among those who currently do not offer telemedical appointments, only 12.7% are planning to offer telemedical appointments in the future. Reasons for not offering telemedical appointments include lack of monetary compensation (28.8%), complicated prescription process (24.2%), and lack of knowledge about telemedical appointments (16.7%). Among those who currently offer telemedical appointments, 41.6% offer them often or very often. Gynecologic oncology (including senology) was the specialty in which telemedical appointments were offered most often (66.7%).

### Future trends and obstacles

**Fig. 2:**
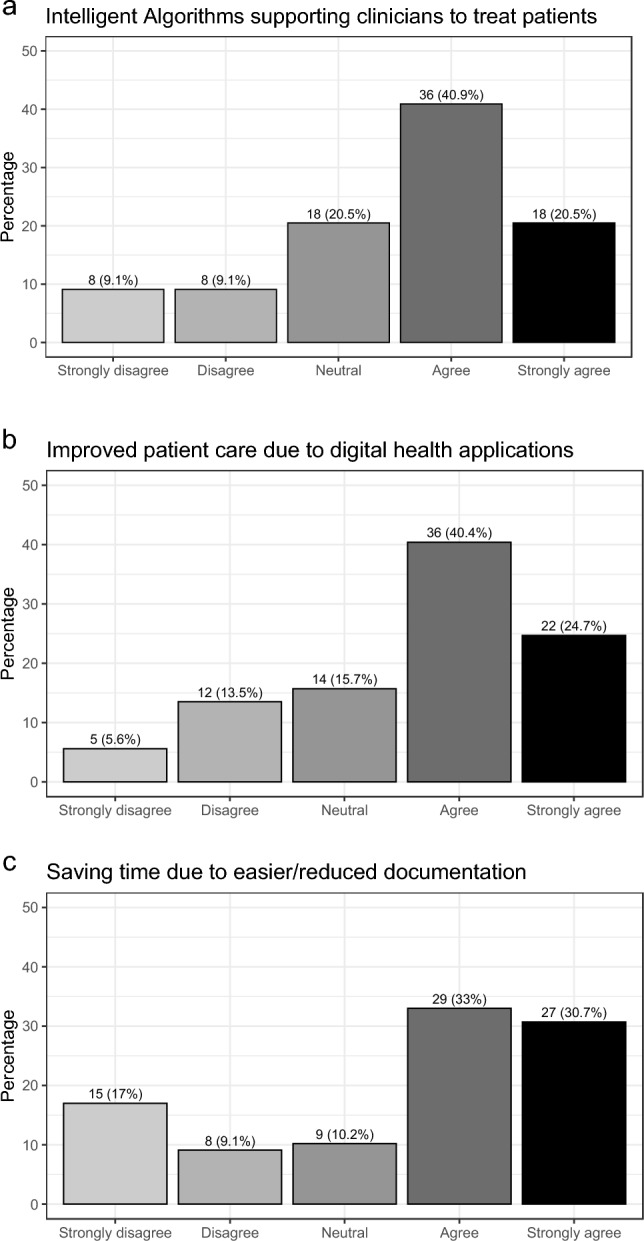
Trends of digital medicine in Germany for gynecology and obstetrics

**Fig. 3:**
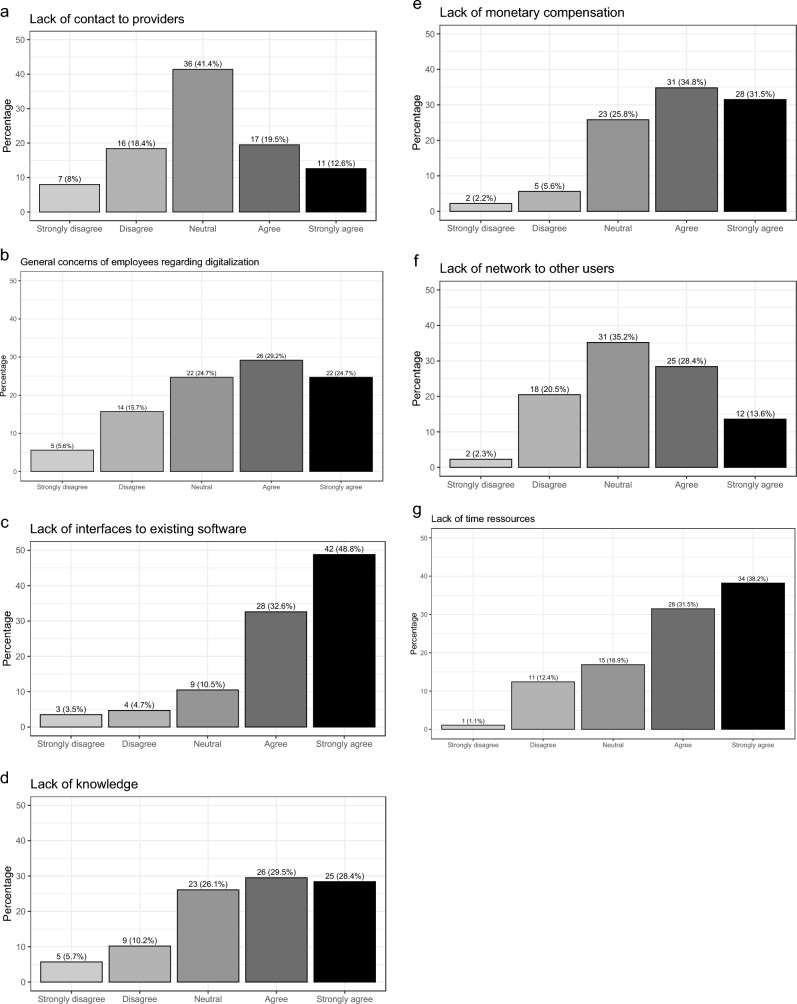
Obstacles of digital medicine in Germany for gynecology and obstetrics

Future trends and obstacles of digital medicine in Germany for gynecology and obstetrics are summarized in Figs. 2 and 3. With respect to future trends, the majority agrees or strongly agrees that digitalization will help save time due to reduced/easier documentation (63.7%), that patient care will be improved by DIGAs (65.1%), and that intelligent algorithms to support clinicians with patient care will be implemented (61.4%).

With respect to obstacles, the majority agrees or strongly agrees that software interfaces to existing software are missing (80.4%), that there is a lack of time to try out new things (69.7%), that there is a lack of knowledge about digital solutions (57.9%), that there is a lack of monetary compensation (66.3%), and that employees have concerns regarding digitalization (53.9%).

## Discussion

In this survey, we aimed to establish the current status quo and future directions of digital medicine for the specialty gynecology and obstetrics in Germany. The majority of survey participants indicated that they believe digitalization can help reduce the increasing workload, improve patient care, and that intelligent algorithms will be implemented to help clinicians provide patient care. Nevertheless, only a small proportion of respondents use digital health applications in their daily routine so far (e.g., DIGAs and telemedicine), and among those who do not already use digital approaches for healthcare, very few are planning to do so in the future. The main obstacles to utilizing digital solutions were a lack of knowledge, insufficient monetary compensation, missing interfaces to existing software, and a complicated prescribing process. These results represent an important call to action.

Despite its tremendous potential, digital health faces numerous challenges. With regard to the lack of knowledge of healthcare professionals, the medical community—especially the digital committee of the DGGG—is called upon to offer appropriate training in the field of digitalization. In our survey, only 13.5% reported receiving training for digital health applications, and 53.9% reported concerns about digitalization among employees. Considering how fundamentally digitalization is changing medicine, there is an urgent need to improve this status. Lack of knowledge represents a major implementation barrier and only an educated, digitally literate medical community can help to shape digitalization in the interest of better patient care [14, 15]. In addition, politicians must create the right framework to encourage practitioners to implement and use digital tools. These conditions include appropriate monetary compensation, the establishment of clear and uncomplicated implementation and prescription processes, as well as technical standards and harmonized interfaces. Still, setting up these standards constitutes a challenge in Germany due to the federal structures that have grown side by side and the resulting, differentiated data structures. The regulatory requirements laid down in the KHZG (Krankenhauszukunftsgesetz, “Future Hospital Law”) may help to achieve these standards [16]. However, hospitals and practices will be confronted with considerable additional financial expenditures [17]. Indeed, lack of financial resources may represent a major barrier, as a series of socioeconomic shocks in recent years have taken a toll on healthcare systems worldwide and introduced several new challenges for the German healthcare system, too [18]. While health spending in the Federal Republic of Germany already rose to an all-time high of €440.6 billion in 2020 (corresponding to 13.6% of the gross domestic product, (GDP), economic stability of German hospitals was maintained through government compensation of €67.9 billion for pandemic-related revenue losses. Despite the highest healthcare expenses per GDP in Europe, the quality of care in Germany is only mediocre compared to other European countries [19, 20]. It is obvious that new paths must be taken to meet the current challenges facing our healthcare system and according to the reform plan “Digitalization Strategy for Healthcare and Nursing” issued by the German Federal Ministry of Health (BMG) in March 2023, digitalization represents an important pillar in this transformation [16].

The gap between high-level evidence and the actual implementation of digital health applications is astonishing. For example, remote symptom monitoring in oncology via electronic patient-reported outcomes (ePROS) showed not only improved patient satisfaction and better quality-of-life but also improved survival in randomized controlled trials [21–23]. Nevertheless, very few of these tools are used in clinical routine, mainly because of the aforementioned barriers. In the field of AI-based decision support tools, a considerable amount of evidence is based on retrospective validation studies with a lack of prospective validation trials. Nevertheless, promising studies exist: for example, AI-supported colposcopy (improved sensitivity for CIN2 + detection of the AI-assisted colposcopy compared to human colposcopists, 96.6% vs. 88.8%, *p* = 0.016) [24], AI-supported response assessment to neoadjuvant systemic treatment in breast cancer (improved sensitivity 100% vs 88% compared to clinical imaging and minimally invasive biopsies to potentially safely eliminate the need for surgery) [25], or AI-supported evaluation of screening mammography (equivalent performance compared to human image readers) [10]. DiGAs are thus already integrated into daily care. They represent scientifically supported, indication-based, and digitally supported care offerings intended to complement guideline-based care and analog interventions. Currently, DiGAs focus on chronic and oncological diseases such as bronchial asthma, diabetes mellitus, or breast cancer. These applications generate valuable data sets that can be used to drive innovative patient care alongside scientific knowledge. In addition, telemedicine is not yet widespread in gynecology and obstetrics, although some studies show that the use of telemedicine also has potential in gynecology and can help ensure effective and comprehensive clinical care for certain indications and reduce the burden on practices and clinics [26]. A relatively new and currently controversially debated application is the use of natural language models, for example, ChatGPT. Just 6 months after its release, its functions are already being discussed in connection with numerous medical applications, ranging from scientific writing and patient conversations to clinical decision support [27, 28]. A recently published study even showed that the chatbot responses to several public questions were preferred over physicians’ answers [29]. For many applications, though, scientifically accepted medical evidence is still lacking. Nevertheless, ChatGPT underlines the urgent need for the medical community to address the matter of digitalization in gynecology and obstetrics from a medical and scientific point of view.

## Conclusion

Digitalization is changing medicine and it is important for physicians to actively shape this process in the interest of female patients. New opportunities for patient communication and treatment should be evaluated and, if appropriate, also implemented and used. The lack of use of digital health approaches in clinical routine as well as skepticism and uncertainty among the gynecologists surveyed shows that there is a great need for education and training on the topic of digitalization. Moreover, a broad-based initiative to evaluate the use of new digital tools such as artificial intelligence and ‘big data’ is also needed in academic research. In the future, the scientific society DGGG is encouraged to take a leading role in implementing digital health, from research to direct patient care and outcome research. By improving the efficiency and quality of treatment directly oriented to the needs of patients, acceptance among physicians should also increase, rendering digitalization in gynecology and obstetrics a success for all involved.

## Appendix: Kommission Digitale Medizin, Deutsche Gesellschaft für Gynäkologie und Gebursthilfe (DGGG)

Alexander Hein, MD

André Pfob, MD

Andreas Hartkopf, MD

Andreas Schmutzler, MD

Armin Wöckel, MD

Bernadette Jäger, MD

Christian Bayer, MD

Christoph Hillen, MD

Claus Richard Lattrich, MD

Dominik Dannehl, MD

Elke Schulmeyer, MD

Elsa Hollatz-Galuschki, MD

Eric Steiner, MD

Fran Kainer, MD

Hanna Hübner, MD

Harald Abele, MD

Heike Janse, MD

Jan Philipp Cieslik, MD

Jan Weichert, MD

Katharina Seitz, MD

Lea Louise Volmer, MD

Maike Henninsen, MD

Maria M Karsten, MD

Marion Kiechle, MD

Markus Wallwiener, MD

Martin Hirsch, MD

Martin Weiß, MD

Matthias Alexa, MD

Max Hackelöer, MD

Oliver Graupner, MD

Peter Fasching, MD

Sascha Hoffmann, MD

Sebastian Griewing, MD

Stephanie Wallwiener, MD

Sven Becker, MD

Tanja Fehm, MD

Thomas Deutsch, MD

Uwe Wagner, MD

## Abbreviations

CI: Confidence interval
IT: Information technology
SD: Standard deviation
